# Severe ectopic Cushing syndrome in a transgender man with a metastatic gastrinoma and an adrenal tumor—A case report and review of the literature

**DOI:** 10.3389/fendo.2023.1135016

**Published:** 2023-03-16

**Authors:** Arnika Wydra, Karolina Cylke-Falkowska, Izabella Czajka-Oraniec, Agnieszka Kolasińska-Ćwikła, Jarosław Ćwikła, Wojciech Zgliczyński, Maria Stelmachowska-Banaś

**Affiliations:** ^1^Department of Endocrinology, Centre of Postgraduate Medical Education, Warsaw, Poland; ^2^I Department of Internal Medicine, Bielański Hospital, Warsaw, Poland; ^3^Department of Oncology and Radiotherapy, Maria Skłodowska-Curie Memorial Cancer Center, Warsaw, Poland; ^4^Department of Radiology, Maria Skłodowska-Curie Memorial Cancer Center, Warsaw, Poland; ^5^Department of Cardiology and Internal Medicine, Medical School University of Warmia and Mazury, Olsztyn, Poland; ^6^Diagnostic and Therapy Center – Gammed, Warsaw, Poland

**Keywords:** pancreatic neuroendocrine neoplasm, ectopic Cushing syndrome, adrenal adenoma, transgender, gastrinoma

## Abstract

A 38-year-old transgender man with advanced metastatic functional pancreatic neuroendocrine neoplasm (PanNEN) gastrinoma was admitted to the Department of Endocrinology due to severe ACTH-dependent hypercortisolemia. An ectopic production of ACTH by PanNEN was suspected. The patient qualified for bilateral adrenalectomy after preoperative treatment with metyrapone. Finally, the patient underwent resection of the left adrenal gland with the tumor only, which surprisingly resulted in a significant decrease in ACTH and cortisol levels, leading to clinical improvement. Pathology report revealed an adenoma of the adrenal cortex with positive ACTH staining. The result of the simultaneous liver lesion biopsy confirmed a metastatic NEN G2 with positive ACTH immunostaining as well. We looked for a correlation between gender-affirming hormone treatment and the onset of the disease and its rapid progression. This may be the first case describing the coexistence of gastrinoma and ectopic Cushing disease in a transsexual patient.

## Introduction

Gastrinoma is a functional neuroendocrine neoplasm (NEN) most commonly located in the duodenum (70%) and the pancreas (25%), and rarely in other locations (5% in the stomach, liver, ovary, and lungs) (1, 2). Its main characteristic is secreting gastrin, which causes clinical signs and symptoms of Zollinger–Ellison syndrome (ZES). In 20%–25% of cases, it occurs as a part of the multiple endocrine neoplasia type 1 syndrome (MEN1), which is an autosomal dominant disorder (3, 4).

ACTH-secreting pancreatic NENs (PanNENs) are rare. They are responsible for up to 15% of ectopic Cushing syndrome cases and result from unregulated ACTH expression and secretion, which significantly worsens the disease prognosis (5). Additionally, ectopic Cushing has been described in up to 5% of gastrinoma cases in the literature (6).

We present a unique case of a transgender man diagnosed at the age of 36 with an advanced pancreatic gastrinoma leading to the symptoms of ZES, in whom an ACTH-dependent Cushing syndrome occurred 1 year later.

## Case description

A 37-year-old man treated for PanNEN gastrinoma with multiple liver metastases was referred to our Department of Endocrinology in May 2021 due to severe epigastric pain and a suspicion of interstitial lung disease on everolimus therapy.

The patient’s medical history included Hashimoto’s disease, nephrolithiasis, and chronic gender-affirming hormone treatment with Testosteronum prolongatum 100 mg every 4 weeks i.m. The patient was transgender, and at the age of 28 years, he underwent female-to-male surgery, including breast reduction surgery, hysterectomy, and bilateral salpingo-oophorectomy.

**Figure 1:**
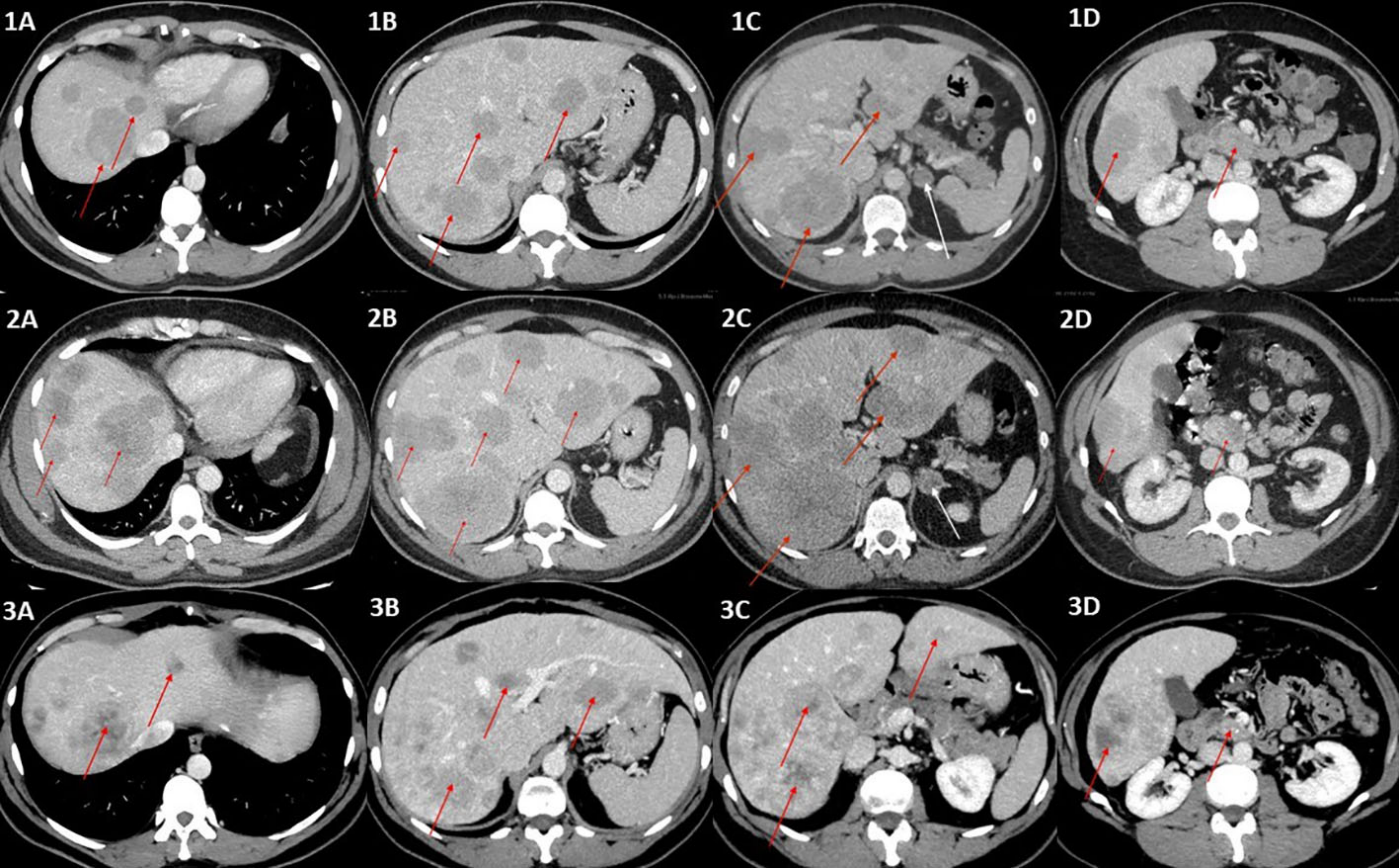
**(1A–1D)** Contrast-enhanced CT scans of the abdomen (portal–venous phase) showing multiple metastases in the liver, ranging in size from a few millimeters to 40 mm (red arrows), incidentaloma of the left adrenal gland of benign phenotype 20 mm in diameter (white arrow), and primary tumor (red arrow) **(1D)**. **(2A–2D)** Contrast-enhanced CT (portal–venous phase) scans revealed progression of the metastatic liver disease (red arrows) and a significant change in the left adrenal tumor density (47 HU in native phase) and size (24 × 18mm), arousing suspicion of adrenal metastasis (white arrow), which was not confirmed in final pathology reports. The primary tumor is indicated by a red arrow in **(2D)**. **(3A–3D)** Post left-sided adrenalectomy contrast-enhanced CT scan showing stable metastatic liver disease, without further progression in terms of intensity, number, and size of metastases, and even with some of the hepatic metastases shrinking (red arrows). The primary tumor is indicated by a red arrow in **(3D)**.

**Figure 2:**
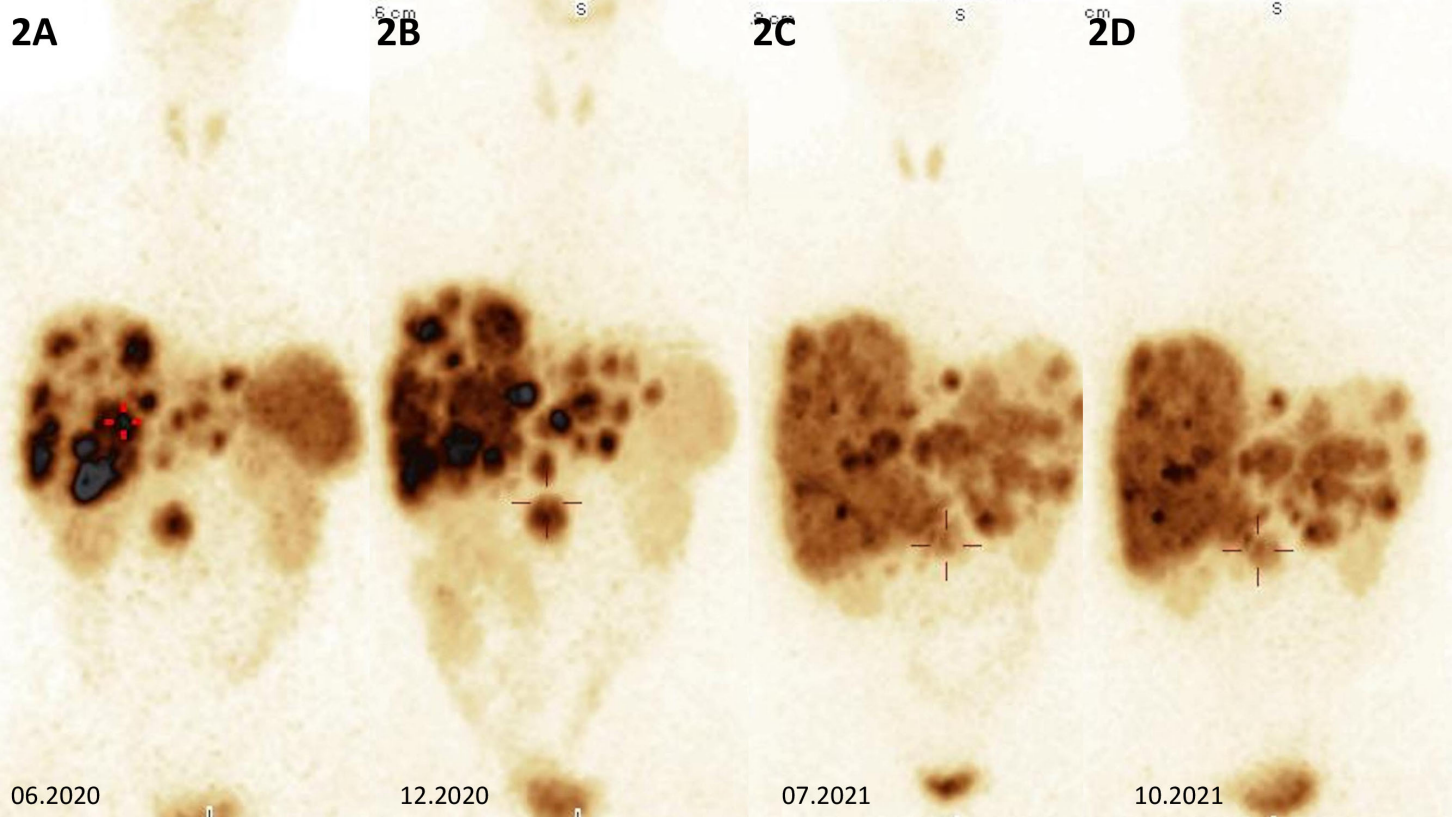
**(A)** Somatostatin receptor scintigraphy of the abdomen showing multiple metastases in the liver, primary tumor in the head of the pancreas with high somatostatin receptor expression in all lesions, and incidentaloma of the left adrenal gland without pathological radiolabel uptake. **(B, C)** Somatostatin receptor scintigraphy showing metabolically receptor-mediated progression within the liver and primary tumor of the pancreatic head. **(D)** Somatostatin receptor scintigraphy showing metabolic receptor-mediated stabilization within metastatic lesions in the liver, with a decrease in liver size and a primary tumor of the head of the pancreas with similar radioimmunoassay accumulation after CAPTEM therapy.

He was diagnosed with gastrinoma in 2020 following 1 year history of heartburn and epigastric pain. In 2019, repeated gastroscopies were performed, revealing recurrent and chronic inflammatory lesions in the esophagus, gastric mucosa, and duodenum. The *Helicobacter pylori* test was positive, and the infection was treated with no improvement. Additionally, abdominal ultrasound showed numerous lesions described then as presumably hemangiomas of the liver, which were later visualized in computed tomography (CT). He also had a 16-mm tumor of the left adrenal gland, which was first diagnosed in 2018 and verified at that time as a non-functioning adenoma with a benign imaging phenotype in CT [3 Hounsfield Units (HU) in the native phase]. In 2020, a follow-up abdominal CT scan showed a 27 × 34 mm pancreatic uncinate tumor infiltrating the superior mesenteric vein and multiple hypervascular metastases in the liver. There were nearly 30 lesions, which ranged in size from a few millimeters to 40 mm (**Figures 1, 1A–D**). A percutaneous liver biopsy was performed and immunohistochemical (IHC) staining revealed the following: NET G2, synaptophysin (+++), chromogranin A (+), and CD 56 (−) with a Ki-67 labeling index of 4%–5%. Somatostatin receptor scintigraphy (SRS) ([^99m^Tc] HYNIC-TOC Tektrotyd, Polatom, PL) subsequently confirmed a pathological radiolabel uptake in the pancreatic and liver lesions presented in **Figure 2**, during initial SRS in June 2020 (**Figure 2A**). There was no pathological radiotracer accumulation in the left adrenal gland, indicating benign adenoma morphology. Laboratory tests revealed inappropriately increased gastrin levels, confirming the diagnosis of gastrinoma. Considering the advanced disease stage, the patient was disqualified from surgical intervention. He was treated at The Maria Sklodowska-Curie National Research Institute of Oncology Warsaw with somatostatin receptor ligand (SRL, octreotide LAR 30 mg every 4 weeks), and then everolimus (10 mg p.o. per day) was started in February 2021 due to progression in the liver.

At the first admission to our Department in 2021, the chest and abdominal CT scans revealed further disease progression with multiple possible metastases in the lungs and a remarkable change in left adrenal tumor density up to 47 HU in the native phase and in size of up to 24 × 18 mm, arousing suspicion of adrenal metastasis (**Figures 1, 2A–D**). His laboratory tests showed highly elevated gastrin and chromogranin A levels during proton pump inhibitor (PPI) and famotidine treatment, elevated PTH level, normocalcemia, and normophosphatemia with vitamin D deficiency. However, genetic testing for MEN1 syndrome, which was performed earlier, was negative. PTH levels normalized after vitamin D deficiency treatment.

Everolimus treatment was discontinued as its adverse effect in the form of nonspecific interstitial pneumonia was suspected. Later imaging studies ruled out lung metastatic lesions.

**Figure 3:**
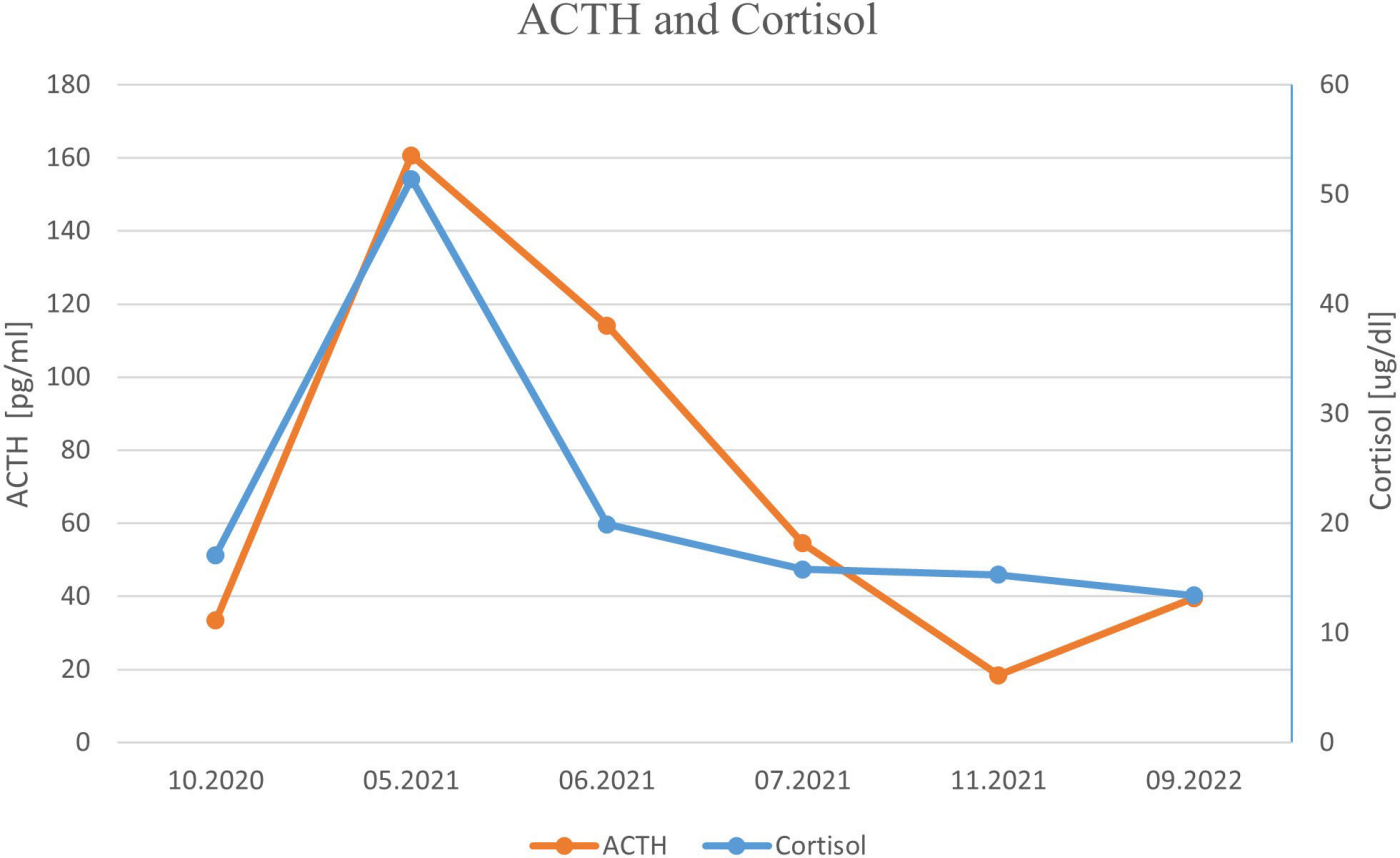
ACTH and cortisol levels presented in chronological order.

One month later, the patient was readmitted to our Department due to a sudden onset of severe hypercortisolemia, leading to severe hypokalemia (**Table 1**; **Figure 3**), increased peripheral edema, and hypertension. He presented some features of Cushing phenotype with easy bruising, weight gain, and severe psychotic symptoms and memory impairment. Hormonal results confirmed the diagnosis of ACTH-dependent Cushing syndrome most likely due to ectopic ACTH or CRH secretion by PanNEN. Head and pituitary magnetic resonance imaging (MRI) excluded the presence of brain metastases and a pituitary adenoma. Metyrapone treatment in a dose up to 2 g per day and pasireotide s.c. 600 μg bid were promptly initiated together with potassium supplementation, spironolactone, and hyperglycemia treatment, which resulted in significant clinical and biochemical improvement (**Table 1**; **Figure 3**). We considered osilodrostat treatment, but it was not available at the time. The patient qualified for bilateral adrenalectomy to treat hypercortisolemia permanently, as the primary PanNEN was inoperable. He underwent surgery after achieving stabilization of his clinical condition. However, due to intraoperative conditions (significantly enlarged liver), the patient underwent left-sided adrenalectomy only. A pathological report revealed an adenoma of the adrenal cortex: chromogranin A (−), inhibin (+), melanin A (+), and a Ki-67 labeling index of 3%–4%, with positive staining for ACTH (+). Liver lesion samples, which were also taken intraoperatively, showed NET G2 with synaptophysin (+++), ACTH (+), and a Ki-67 labeling index of 7%–8%. After adrenalectomy, hormonal results revealed a significant decrease in ACTH level, normal morning cortisol levels, and 24-h free urinary cortisol excretion, and a further increase in gastrin concentration (**Table 1**; **Figure 3**). Owing to the progression of liver metastatic lesions described in the abdominal CT and SRS and increasing gastrin and CgA levels, the patient qualified for temozolomide and capecitabine chemotherapy (with standard dosing capecitabine 750 mg/m^2^ bid on days 1–14 and temozolomide 150 mg/m^2^ p.o. on days 10–14), while continuing SRL therapy, with a good initial response in the form of a decrease in the mass of metastases and gastrin level. However, chemotherapy with temozolomide and capecitabine was discontinued after the fourth course due to recurrent severe pancytopenia with life-threatening anemia requiring multiple blood transfusions. During a follow-up hospitalization, laboratory results confirmed prolonged normalization of ACTH and cortisol levels (**Table 1**; **Figure 3**). 18F−fluorodeoxyglucose PET-CT imaging ruled out the presence of metastases in the lungs and confirmed the metabolically active process located in the pancreatic head and in the liver.

**Table 1:** Laboratory results presented in chronological order.

Parameter	Normal range	Baseline results (October 2020)	During hypercortisolemia (May 2021)	During metyrapone treatment (June 2021)	After adrenalectomy (July 2021)	During chemiotherapy (November 2021)	Currently (September 2022)
Gastrin [pg/ml]	13–115	2,872	15,376	26,150	60,137	14,596	11,224
Cg A [ng/ml]	0–100	>1,050	>1,050	>1,050	>1,050	613.9	865
Cortisol [μg/dl]	5–22	17.1	51.6	19.9	15.8	15.3	13.4
ACTH [pg/ml]	7.2–63.3	33.4	160.7	83.9	54.5	18.4	39.6
Potassium [mmol/L]	3.5–5.1	3.28	3.22	4.68	4.48	4.09	4.64
UFC [μg/24 h]	20–130.9	75.6	2009	782	70.6	114	128

In the latest CT performed in August 2022, 9 months after the end of temozolomide and capecitabine chemotherapy, the disease was stable, without further progression in terms of intensity, number, and size of metastases, and even with some of the hepatic metastases shrinking (**Figure 1**).

Currently, 1 year after adrenalectomy, we found stable, slightly elevated levels of morning ACTH, normal cortisol with maintained diurnal rhythm, 24-h free urinary cortisol excretion in the upper limit of normal, testosterone concentration within the male range, and elevated but stable gastrin and chromogranin A levels (**Table 1**; **Figure 3**). The patient showed declining ZES symptoms during PPI treatment.

## Discussion

Gastrinoma is a rare functional NEN (0.5–21.5 cases per million persons annually) that occurs sporadically in 75%–80% of cases (3, 4). Its cells produce excessive amounts of gastrin, leading to the fundic parietal cell hyperplasia and a significant increase in basal gastric acid secretion and, as a result, peptic ulcer disease, advanced gastroesophageal reflux, and chronic diarrhea, which is called ZES (2, 7, 8). Diagnosis of ZES is often a challenge because hypergastrinemia might result from several different causes, such as *H. pylori* infection, extensive small-bowel resection, gastritis, PPI, or high-dose histamine H2-receptor antagonist treatment, vagotomy, chronic renal failure, and atrophic gastritis (1). Chromogranin A is a nonspecific biomarker, though it may provide significant prognostic information and is often used in long-term follow-up. Although gastrinomas are well-differentiated NENs G1/G2, these tumors are malignant in 60%–90% of cases (2, 8). It is worth emphasizing that despite the fact that both duodenal and pancreatic gastrinomas commonly involve lymph nodes (30%–70%) (8–11), pancreatic tumors are more frequently associated with liver metastases (10, 11).

Additionally, an ectopic ACTH secretion by a malignant gastrinoma is rarely described.

The earliest reports on the co-occurrence of gastrinoma and ectopic Cushing’s syndrome were described by Del Castillo et al. who described in detail the occurrence of Cushing’s syndrome 1–3 years after the diagnosis of ZES (12). In our case, a similar interval was observed between the diagnosis of ZES and hypercortisolemia. Both reports include patients with metastatic liver lesions, which are associated with worse prognosis and shorter life expectancy (13). We also observed multiple metastatic liver lesions and a sample taken from such a lesion had positive staining for ACTH on histopathological examination. The development of Cushing’s syndrome is an adverse prognostic factor. Yu et al. reported that survival rate in gastrinoma patients after ectopic ACTH secretion diagnosis averages 1.7 years (11). We did not observe in our patient any disease progression after 2 years since the onset of Cushing syndrome. However, he was successfully treated from hypercortisolemia with adrenalectomy and additionally highly effective chemotherapy with temozolomide and capecitabine was introduced following Cushing syndrome resolution.

Castro et al. described a series of nine cases diagnosed with ectopic Cushing’s syndrome, with a surprisingly high co-incidence of gastrinoma of 33%, compared with other series in which such a diagnosis was made in 5% (6). However, the authors related these discrepancies to the large impact of randomization in the small study group (14).

As mentioned before, ZES may be a manifestation of MEN1 together with a parathyroid adenoma (90%) and pituitary adenoma (30%–40%) (12). MEN1 diagnosis should be considered especially in young adults (32–35 vs. 48–55 years of age in sporadic type) and patients with a positive family history. In MEN1, the pancreaticoduodenal tumors have a set of unique features, and apart from an earlier age of onset, these include preneoplastic hyperplasia and multiple microadenomas diffused throughout the affected tissue, which precedes the development of clinically significant tumors (15).

In the case of our patient, genetic testing did not confirm *MEN1* mutation, the suggestion of which arose not only from the patient’s young age but also from elevated PTH levels. However, increased PTH with normocalcemia was presumably caused by vitamin D deficiency in our patient and it later normalized upon proper treatment. In addition, primary hyperparathyroidism should have been ruled out as a possible reason for the increase in gastrin levels (16).

Our patient is a transgender person and, as such, has probably a different cancer risk. Transgender patients have been described to have increased exposure to sexually transmitted diseases and some well-known cancer risk factors, such as smoking and alcohol use. Cancer incidence in transgender people has been identified as an important priority for further research (17). Data on cancer prevalence and mortality in this group are lacking, although overall mortality is certainly higher compared with the general population (17, 18). According to available literature, transgender men have an increased risk of breast, cervical, lung, hematological, gastrointestinal, liver, and pancreatic cancers, brain tumors, and melanomas (17, 19). There was one case of pancreatic mucinous cystic neoplasm described in a transgender female-to-male patient (20). To our knowledge, no case of PanNEN in a transgender patient has been reported to date. There is evidence that pancreatic tissue expresses estrogen and androgen receptors, but the role of transgender chronic testosterone therapy in pancreatic carcinogenesis remains unclear (20). Qui et al. demonstrated an association between estrogen concentrations and the course of PanNEN, showing a better prognosis in terms of tumor size and survival time in patients with higher estrogen concentrations. The authors emphasize the protective effect of estrogen against PanNEN development (21). In addition, higher estrogen receptor beta expression proved to be a favorable prognostic indicator in PanNEN (22). Moreover, men have a higher rate of disease post-resection recurrence for well-differentiated PanNEN, and they are at higher risk of complications, such as pancreatic fistula (23, 24). The highlighted issues of sex-related differences in PanNEN require further exploration with specifically tailored real-life studies. Our patient was on permanent testosterone replacement after a female-to-male sex change.

In general, the gender-affirming hormone treatment may affect not only the risk for hormone-dependent sex-specific cancers but also other types of neoplasms that may express sex hormone receptors. Thus, the question of long-term sex hormone gender-affirming treatment influence on cancer risk remains.

In gastrinoma, surgical intervention is the only curative option, and it is the treatment of choice whenever feasible. Medical management with high doses of PPIs and H2 antagonists (three to four times the standard dose) is used to control the hydrochloric acid hypersecretion, thus alleviating symptoms and preventing complications, such as intestinal bleeding or perforation from duodenal or gastric ulcers. The patients who are not qualified for surgical intervention receive long-term PPI treatment (2, 25). Often, even after successful gastrinoma resection, gastric acid hypersecretion persists, and continuation of PPIs is necessary (26). Upfront surgery should be excluded in the presence of extra-abdominal metastases and high-grade PanNENs. Additionally, liver transplantation may be considered if the patient with numerous liver metastases meets specific criteria. In patients with liver metastases who are disqualified from complete resection, vascular and ablative locoregional treatment may be considered. In our patient with the advanced stage of the somatostatin receptor (SSTR)-positive G2 PanNEN with multiple liver metastases and persisting ZES symptoms, despite continuous PPI treatment, SRL was initiated with the later addition of everolimus. Unfortunately, non-specific interstitial pneumonia soon followed, an adverse effect of everolimus present in approximately 12%–16% of treated patients (27). Chemotherapy or peptide receptor radionuclide therapy (PRRT) was initially not implemented due to the rapid onset of severe hypercortisolemia.

ACTH-secreting PanNENs are rare (5). In some of the described cases, ZES did not occur simultaneously with Cushing syndrome, meaning that with the progression of the disease, neoplastic cells acquired the ability to secrete ACTH (28). Additionally, Cushing syndrome in patients with ZES can be fatal even in the absence of NEN progression due to complications of hypercortisolemia and comorbidities (5).

Cushing syndrome is associated with psychiatric and neurocognitive disorders, with depression, anxiety, and neurocognitive impairment being the most common (29). True melancholic syndromes, refractory to standard treatment, are also possible. As in the case of other complications, achieving control of cortisol excessive secretion is the most effective management. For successful treatment planning, it is crucial to find an exact cause of ACTH-dependent hypercortisolemia. If the source of ACTH overproduction is uncertain or inoperable, the only rescue treatment might be bilateral adrenalectomy.

In our patient, a head MRI was performed and metastases to the central nervous system and lesions of the pituitary gland were excluded. Abdomen CT showed NEN progression and an enlarged adrenal tumor, which could have been also a metastasis or plausibly a lesion responsible for hypercortisolemia. Kanakis et al. showed no increased incidence of NEN metastases to adrenal glands. In a group of 438 patients, they found only one such case (30). Falhammar et al. reviewed 164 cases of Cushing’s syndrome and 77 cases of pheochromocytomas and found only two cases of ectopic ACTH production from adrenal medullary lesions (one case of pheochromocytoma and one case of adrenal medullary hyperplasia) (31). Moreover, Hiroi et al. described a case of an adrenocortical–pituitary hybrid adrenal tumor, which was the source of ectopic ACTH production. The above-mentioned tumor consisted of cells typical for adrenocortical adenoma, but with immunoreactivity to ACTH and synaptophysin. Further analysis confirmed the presence of elements characteristic of both steroid-producing cells and typical corticotroph secretory granules (32). There have also been several cases of ACTH-secreting pheochromocytomas (33, 34). Although they are very rare, all the above possibilities had to be considered in our case.

Considering the severe state of our patient, no dynamic hormonal testing was performed to confirm ectopic ACTH secretion. Pharmacological treatment with metyrapone was promptly initiated. The patient was referred to bilateral adrenalectomy; however, due to technical problems, he underwent left-sided adrenalectomy only. Nevertheless, normocortisolemia was achieved, and it persisted through to the 1-year follow-up. What is more surprising, the level of ACTH decreased directly after surgery, suggesting the adrenal source of its oversecretion, which may have been related to the hormonal activity of the adrenal adenoma that was confirmed by positive staining for ACTH. Additionally, it may be correlated to the removal of liver metastases, which could be responsible for ectopic Cushing’s syndrome. However, during surgery, only small samples of liver tumors were taken for histopathological evaluation. The cyclic ectopic production of ACTH by metastatic PanNEN cannot be completely ruled out in our case as well.

Chemotherapy is the next line of treatment for PanNEN; therefore, in our patient, capecitabine with temozolomide was initiated considering the results of Kuntz et al. (32). The treatment led to significant improvement and a decrease in liver metastasis sizes. It may have been an important factor in maintaining normocortisolemia and the normal level of ACTH as its expression in liver lesions was also confirmed. These two approaches should be taken into consideration in explaining the cause of ectopic Cushing’s syndrome in our patient. Now, the patient is still managed with SRL, due to the high expression of SSTR in functional testing. Follow-up examination shows normal levels of ACTH and cortisol, reduction of the primary tumor size, and metastatic lesions in the liver. Additionally, the patient is continuing gender-affirming hormone treatment with testosterone. PRRT, surgical cytoreduction, and continuation of chemotherapy are the next management option in case further progression occurs.

## Conclusions

Gastrinomas are rare and often malignant functional NENs. Despite the initial confirmation of NEN’s secretory profile, new hormonal activity may appear in the course of the disease. It could be caused by a change in either primary or metastatic tumors’ secretion, or it could be due to a completely new disease manifestation. Such complication may worsen the patient’s prognosis, and it may require a multimodal approach and the involvement of different specialists in diagnostic and therapeutic processes. In young adults with NENs, MEN1 syndrome should be considered in the differential diagnosis, because its management differs significantly. In view of the continuing increase in the number of transgender people, further study of the impact of gender-affirming hormone treatment on tumor development is required.

## Patient’s perspective

Currently, the patient reports a significant improvement in his wellbeing and quality of life despite his advanced malignant disease. He states that he would undergo further necessary treatment and he has confidence in the medical care.

## Data availability statement

The original contributions presented in the study are included in the article/supplementary material. Further inquiries can be directed to the corresponding author.

## Ethics statement

Written informed consent was obtained from the individual(s) for the publication of any potentially identifiable images or data included in this article.

## Author contributions

Surgical and Medical Practices: AW, KC-F, IC-O, AK-A, JA, WZ, MS-B. Concept: MS-B, IC-O, AW, KC-F. Data Collection or Processing: AW, KC-F, IC-O, AK-A, JA, WZ, MS-B. Analysis or interpretation: AW, KC-F, IC-O, MS-B. Literature Search: AW, KC-F, IC-O, MS-B. Writing: AW, KC-F, IC-O, MS-B. All authors contributed to the article and approved the submitted version.
